# Meibomian Gland Dysfunction in Ocular Graft vs. Host Disease: A Need for Pre-Clinical Models and Deeper Insights

**DOI:** 10.3390/ijms22073516

**Published:** 2021-03-29

**Authors:** Eugene Appenteng Osae, Philipp Steven

**Affiliations:** 1 College of Optometry, University of Houston, Houston, TX 77004, USA; 2 Department of Ophthalmology, Division for Dry-Eye and Ocular GVHD, Medical Faculty, University of Cologne, 50923 Cologne, Germany; philipp.steven@uk-koeln.de

**Keywords:** ocular graft-vs-host disease, meibomian glands, pre-clinical models, inflammation

## Abstract

Despite decades of experience with hematopoietic stem cell transplantation, we are still faced with the delicate equipoise of achieving stable ocular health post-transplantation. This is because ocular graft-versus-host disease (oGvHD) following hematopoietic stem cell transplantation frequently occurs (≥50%) among transplant patients. To date, our understanding of the pathophysiology of oGvHD especially the involvement of the meibomian gland is still limited as a result of a lack of suitable preclinical models among other. Herein, the current state of the etiology and, pathophysiology of oGvHD based on existing pre-clinical models are reviewed. The need for additional pre-clinical models and knowledge about the involvement of the meibomian glands in oGvHD are emphasized.

## 1. Introduction

Allogeneic hematopoietic stem cell transplantation (aHSCT) from human leukocyte antigen (HLA) matched donors (related or unrelated) is widely used as treatment for a myriad of hematological diseases, autoimmune diseases and inherited metabolic disorders [1,2,3]. However, a common therapeutic limitation to the success of allo-HSCT is the development of graft-versus-host disease (GvHD). In general, GvHD involves an exaggerated donor-derived lymphocytic reaction to host antigens following aHSTC. These host antigens are called minor histocompatibility antigens (MiHA) and are not included in routine HLA typing as part of the attempts to optimize the outcome of aHSCT [4].

As a multisystem condition, GvHD can affect the skin, gastrointestinal system, the liver, the lungs, oral mucosa, the eye and other organs [3,5,6]. GvHD in the eye is broadly termed ocular graft-versus-host disease (oGvHD), which can affect over 50% of individuals who undergo aHSCT [6,7]. Ocular GVHD may affect all tissues of the eye, however, presents predominantly as disease of the ocular surface including fast progressing inflammation and destruction of the entire lacrimal functional unit leading to severe ocular surface damage [7,8,9,10]. Although often termed a subform of dry-eye disease, oGVHD features distinct differences, such as affection of retina and optic nerve as stated above. Ocular GVHD has serious impact on the quality of life as well as imposes a significant threat of visual impairment due to its rapid and aggressive progression in many cases. Ocular GvHD can exist as acute, chronic, or overlapping forms based on the time of onset post- aHSCT, or the specific organs types involved. The most common form is chronic oGvHD which is accompanied by symptoms such as ocular discomfort, irritation, photophobia, redness, itchiness, foreign body sensation, burning, watery eyes and blurred vision [3,8].

These hallmarks of oGvHD makes the condition mimic other immune conditions such as Sjogren’s or non-Sjogren-associated dry eye, making it clinically challenging to establish definitive diagnosis for oGvHD. In fact, the pathology of oGvHD is very complex and not fully understood. Some pre-clinical studies suggest the pathomechanism of oGvHD may involve the renin-angiotensin system and organelle-level stress signaling mechanisms such as with the endoplasmic reticulum and ultimate cell death [11]. Other studies suggest that a shift in the gut-ocular surface microbiome axis is involved in the development of oGvHD [3,12]. Moreover, some studies suggest the severity of oGvHD is significantly modulated by pre-existing disease conditions or pre-aHSCT conditioning procedures, i.e., immunoablative chemotherapy and/or radiation [3,6].

Most importantly, there is the suggestion that it comprises at least three main biological processes; lacrimal gland dysfunction, corneo-conjunctival inflammation, and meibomian gland dysfunction (MGD) [7,11]. The former two processes have been well studied but little information is known about the latter, that is the involvement of the meibomian glands and their dysfunction thereof in the development of oGvHD [7,13,14,15]. This is partly due to the lack of suitable pre-clinical animal models and sensitive strategies or approaches to study the condition [11,16,17,18,19,20,21,22]. In this review we summarize and present information on the current of state of oGvHD with emphasis on pre-clinical models. A major focus is placed on the knowledge gap about the involvement of the meibomian gland, both as a potential immuno-inflammatory target and perpetuator of disease in oGvHD. We further discuss the need for suitable animal models that better mimic the human oGvHD condition and suggest study strategies that can enhance our understanding of the involvement of the meibomian glands in oGvHD [7].

## 2. Overview of Meibomian Gland Biology

The meibomian glands also called the tarsal glands are specialized lipid-secreting glands located in the upper and lower eyelids. They produce meibum, the tear film lipid component which is crucial in maintaining ocular surface homeostasis by retarding tear film evaporation and preventing dry eye. Meibum is also thought to aid in the prevention of infection at the ocular surface by way of its broad-spectrum antimicrobial properties [23,24].

Given these important functions of meibum, several studies have established that MGD which is characterized by a chronic diffuse abnormality of the meibomian gland is a leading cause of dry eye disease [25,26].

In terms of morphogenesis, the meibomian glands as modified sebaceous glands follow similar developmental code just like the skin sebaceous glands. In fact, the meibomian glands are often regarded as hair follicles without hair shafts [25]. The glands grow from the mesoderm within the third to seventh month of gestation [25,27]. When the ectodermal lens placode invaginates from the lens vesicle, mesodermal loose connective tissue in the lid folds, this differentiates into several structures including the orbicularis and Riolan muscles of the eyelids, blood vessels, underlying areolar tissue of the outer eye lid skin, the conjunctiva and the thin elongated plates of dense connective tissue that forms the tarsal plates [27,28]. Mesenchymal tissue then morphs into the eye folds, creating the palpebral fissure [25].

The glandular acini where lipids are produced, and ductule-ductal systems develop as lateral outgrowth from the epithelial cords of the meibomian anlages. The production of lipids within the anlages leads to the formation of a central canal that later develops into the central duct [28,29]. This early lipid production by more mature meibomian anlages has been reported to be critical for the differentiation of the upper and lower eyelids during the seventh month of gestation [27]. In contrast, increasing keratinization not lipid production is thought to partly mediate the separation of the upper and lower lids in the mouse [25,27]. In spite of this, some studies have shown that meibomian gland development in the mouse is similar to humans [29]. It is however shorter in relative duration and begins at embryonic day (E) 18.5 with full mature gland morphology observable at post-natal day (P)15 [29].

## 3. Secretory and Postulated Regeneration Properties of the Meibomian Gland

**Figure 1:**
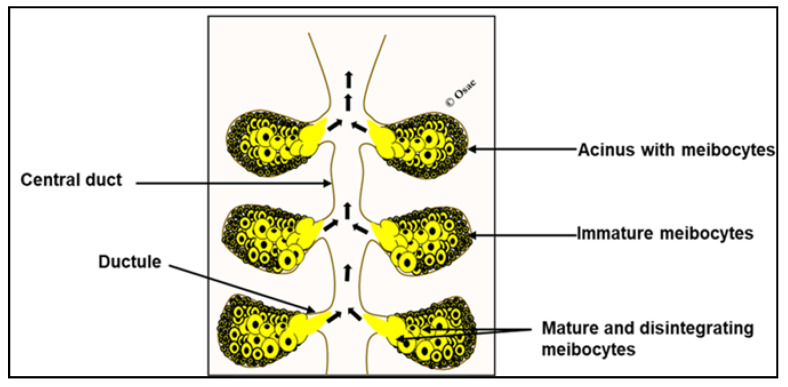
Schematic of the meibomian gland. Immature meibocytes reside at the periphery of acini and mature ones are central. Hyper-mature and lipid-laden meibocytes rapture and release meibum for upward delivery (short arrows) at the ocular surface.

The meibomian glands produce and secret meibum at the ocular surface through a holocrine process [25,29,30]. The meibomian gland acini contain cells called meibocytes which synthesize and accumulate meibum. These acini are connected to ductal systems which are lined by proliferating stratified squamous epithelial cells [29,30]. Shorter ductules which are directly connected to the acini are in turn connected to a central duct that extends to the meibomian gland orifice at the eyelid margin [25]. Meibocytes in the acini are organized such that small, immature ones are located at the periphery of the acini and mature ones in the center of the acini [25], (Figure 1). The immature meibocytes arise from proliferating basal cells that line the acini and as they divide and differentiate into mature lipid accumulating forms, they move centripetally [25,29,30]. At this point, their nuclei become pyknotic and undergo programmed disintegration releasing their content (with ruptured cellular components) into ductules for upward delivery at the ocular surface via the central duct and glandular orifice [29,30,31]. Reports suggest that the whole process of meibocyte differentiation and maturation and release of meibum takes averagely 9 days [29,30].

Since meibogenesis ends with a holocrine secretion process, it has been postulated that the continuous loss of meibocytes is also accompanied by constant regeneration [29,30]. That is, basal acinar cells proliferate to generate new cells to replace lost ones through holocrine secretion. Recent studies suggest that, there are stem-like cells around the circumference of meibomian gland acini [30,32]. In fact, some other studies also suggest that quiescent progenitor cells exist around terminal regions of the ductal epithelium and they are believed to be crucial for the maintaining viable basal acinar cells [30,32]. The theory is that, the meibomian gland like other tissues must contain quiescent progenitor cells or stem-like cells as way to sustain the basal acinar cells throughout life [25]. The implication is that, disease, ageing or potentially adverse conditions such as pre-aHSCT conditioning procedures, i.e., immunoablative chemotherapy and/or radiation can destroy these progenitor cell population, thus setting the stage for MGD development [25].

## 4. Pre-aHSCT Conditioning Regimen Can Affect Normal Meibomian Gland Health

Conditioning regimens preceding aHSCT usually consists of high-dose chemotherapy and/or radiotherapy [33]. These procedures have severe acute and delayed toxic effects on several tissues [33,34]. Some studies implicate increased levels of reactive oxygen species and the depletion or exhaustion of antioxidants due to the pre-conditioning procedures as the cause of tissue damage. In one such study, levels of erythrocyte and plasma malondialdehyde (MDA) were significantly elevated after high-dose chemotherapy whereas catalases, superoxide dismutase, glutathione S-transferase levels were decreased [33]. The meibomian gland may not be exempted when it comes to oxidative stress-related tissue damage. In fact, several studies have postulated the potential role of oxidative stress in ocular surface disease [35,36,37], but what remains to be determined is whether these pre-conditioning regimens could elicit a disruption in the pro-oxidative-antioxidant defense system in the meibomian gland leading to its dysfunction and therefore oGvHD [37].

**Figure 2:**
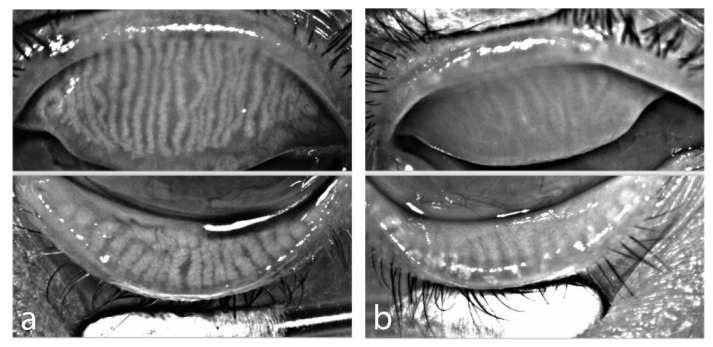
Infrared meibography prior to aHSCT (**a**) OS of female patient, age 58, acute myeloid leukemia with normal distribution of meibomian glands, (**b**) OD of male patient, age 52, chronic myeloid leukemia with extensive loss of meibomian glands in both eyelids (Meibographer: Keratograph 5M, OCULUS Optikgeraete GmbH, Wetzlar, Germany).

Apart from oxidative stress, pre-conditioning chemo-or radiotherapy has other acute or chronic toxic effects that may not spare the meibomian glands. When chemotherapy and radiotherapy are combined, as is often the case with most pre-aHCST treatments, it becomes difficult to differentiate the source of toxicities. However, studies have reported that a major side effect of the chemotherapy is that it not only destroys malignant cells but can destroy normal or healthy rapidly dividing cells types such as the skin cells, hair follicles, germinal cells and others [38]. As previously reported, basal meibomian gland acinar cells undergo a fairly rapid normal cell division and differentiation to replace meibocytes lost through holocrine secretion. These can be destroyed by the chemotherapy just as occurs in other normal rapidly dividing cells [38]. Similarly, radiotherapy can result in unwanted side effects of cataracts, infertility, alopecia and skin toxicities [38,39]. Given the closeness of the developmental biologies of the meibomian gland and the sebaceous glands of the skin, it will not be incorrect to assume that the meibomian glands could be impacted in a similar fashion [25,38]. There is the likelihood that, pre-conditioning radio-or chemo- therapy or both can also deplete meibomian quiescent progenitor cells or stem-like cells postulated to be crucial for its maintenance or regeneration [32,38]. This may initiate meibomian gland atrophy leading to MGD therefore oGvHD [25]. In fact, studies by our group showed, that some patients undergoing aHSCT already demonstrate signs of meibomian gland loss, depicted by infrared meibography, whereas others demonstrate normal Meibomian gland distribution Figure 2 [7].

## 5. Evidence of Inflammatory Immune Response in oGvHD and the Meibomian Gland

In addition to tear film insufficiencies, one hallmark of oGvHD is inflammation. Some clinical and pre-clinical animal studies have showed that inflammation is present in either acute or chronic forms of oGvHD with the severity often highly dependent on the severity of systemic disease [19,40,41]. The cornea, the conjunctiva and lacrimal glands have been reported as primary immunological targets in the eye with immunohistological findings comparable to cutaneous GVHD [40,41]. Specifically, there is recruitment and infiltration of neutrophils, monocytes, macrophages, T-cells (CD3+, CD4+, CD8+) and upregulation of certain inflammatory cytokines including interleukin-6, interferon- γ and tumor necrosis factor-alpha [19,20,42].

The immuno-inflammatory component is poorly understood just like the disease entity itself, but the idea is that, donor- derived alloreactive T lymphocytes may play a key role in the development of oGvHD. Furthermore, potential thymic injury during pre-conditioning regimens and/or acute GVHD may lead to defective central tolerance, aberrant proliferation of autoreactive T (principally CD4+ Th2) and B cells, and autoantibody formation leading to oGvHD [18,20,43]. Therefore, oGvHD may involve both cell-mediated and humoral immunity that leads to infiltration and inflammation of the lacrimal gland, conjunctiva, cornea and limbal tissues [20,42,43]. These inflammatory changes may be accompanied by decrease in the density of conjunctival goblet cells with concomitant reduction in mucin levels, scarring of the lacrimal gland and conjunctiva [18,44]. As a consequence, oGvHD patient may experience dry-eye like symptoms such as itchiness, grittiness, foreign-body sensation, photopia, pain, redness and watery eyes [7].

While inflammation is heavily involved in oGvHD, its effects on only the lacrimal gland, cornea, conjunctiva, limbus have been well documented. However, little is known about exactly how these immuno-inflammatory events target the meibomian gland. Considering the ocular surface as one functional unit it suffices to say that the meibomian gland may be impacted in a similar fashion and further investigations using suitable animal model will be prove useful in elucidating the possible immune-inflammatory consequences of aHSCT on the meibomian gland.

## 6. Pre-Clinical Models of oGvHD and the Meibomian Gland

There are few established pre-clinical animal models of oGvHD [18,19,20,21,22,42,45]. Reports on such models show considerable differences especially in donor-recipient matching and mode of induction of GvHD, therefore differences in oGvHD and outcomes of interest measured. Thus far, these models have been very useful in understanding oGvHD but the inherent lack of uniformity among the models suggest there is need for more suitable models that closely mimic the acute and chronic human oGvHD conditions. The interesting commonality among all these pre-clinical studies is that neither of them directly addresses nor presents in-depth discussions about the involvement of the meibomian gland and its dysfunction in the development of oGvHD in the models. Thus, necessitating future (suitable) models to consider direct investigations of the meibomian gland, its dysfunction and contribution to the development of oGvHD. A summary of relevant studies on pre-clinical models of oGvHD with their key findings is presented in Table 1 [18,19,20,21,22,42,45].

## 7. Discussion

The primary goal of this review is to highlight the need for preclinical models as part of the efforts to understand oGvHD. Most importantly, we also aimed at revealing the gap in knowledge about the direct involvement of the meibomian gland and its dysfunction in oGvHD. From the papers reviewed [18,19,20,21,22,42,45], it is clear that much is known about the effects of aHSCT on the cornea, lacrimal gland, conjunctiva and its related goblet cells in terms of the development of oGvHD. Specifically, the immuno-inflammatory consequences associated of aHSCT on these ocular structures including the recruitment and infiltration of neutrophils, monocytes, macrophages, T-cells (CD3+, CD4+, CD8+) and upregulation of certain inflammatory cytokines including interleukin-6, interferon- γ and tumor necrosis factor-alpha which are known to modulate ocular surface disease.

However, there is little indirect or no evidence on how the meibomian glands might be affected in these models [18,19,20,21,22]. The need to understand in pre-clinical models how the meibomian glands may be involved in the development of oGvHD takes on added significance because the meibomian gland and its secretion are crucial in maintaining ocular surface homeostasis [23]. In fact, dysfunctional meibomian glands account for the over 70% of dry eye syndrome, which is an important feature of oGvHD [25].

What calls attention is that, several clinical studies have already reported meibomian gland dropout in aHSCT patients with oGvHD [7,25,46,47]. Some studies even suggest there is some degree of meibomian gland dropout in patients with hematological disorders, especially those with acute malignancies prior to aHSCT, and this is accompanied by significant dry eye [48,49]. These findings are however mainly dependent on conventional meibography and there is need for more mechanistic insights into how the meibomian glands are involved in oGvHD using suitable pre-clinical animal models and study approaches [25]. For example, it is unclear if like the cornea, lacrimal gland, and the conjunctiva, the meibomian gland may also be a direct target of immuno-inflammation events, i.e., recruitment and infiltration of immune cells and inflammatory cytokines following aHSCT. By such, pre-existing MGD and exposure of potential antigen could be a risk factor for later oGVHD. While inflammation of the meibomian gland is a highly debated subject, its occurrence in aHSCT is not precluded given that the ocular surface is a continuous anatomical and functional unit and what happens to the cornea, conjunctiva and lacrimal glands may likely affect the meibomian glands and vice versa. This can lead to MGD which can lead to ocular surface disease that feed into and perpetuate oGvHD [9,10,50,51].

Another important question regarding meibomian glands or MGD involvement in oGVHD is whether pre-conditioning radiotherapy and/or chemotherapy can vaguely destroy its normal quiescent progenitor or stem like cells, depriving the meibomian glands of their ability to replenish lost cells after holocrine secretion [31]. Studies have shown that rapidly dividing normal cells including, germinal cells and skin cells can be destroyed by chemo- and or radiotherapy [31,52]. The meibomian gland cells undergo fairly rapid process of cell division and has important the commonalities with skin sebaceous therefore its progenitor cells may be lost in a similar manner [25,52]. This may better explain the observed loss or dropout of meibomian glands seen among clinical oGvHD patients [7,15].

Functionally, study of meibomian gland lipid samples from various pre-clinical models will also be useful in understanding the meibomian glands in their involvement in oGvHD [53,54]. Since MGD is also characterized by specific biochemical/biophysical alterations in meibum, it will be interesting to determine if such alterations exist in pre-clinical oGvHD models and if they do whether specific lipid signatures can provide insights in the development of MGD and oGvHD [55]. To get a comprehensive picture of how the meibomian glands are impacted, it is also important to pursue these questions with consideration for what kind of pre-conditioning regimen or transplantation matching there are, i.e., syngeneic vs. allogeneic and chemotherapy vs. radiotherapy, respectively. This is because GVHD and/or oGvHD is invariably severer in allogeneic transplantation than syngeneic and a combination of chemotherapy and radiotherapy will have more side effects that only one of them on the eye and its adnexae [19,39].

## 8. Conclusions

In summarizing, this review has revealed there is a need for deeper insights into the pathomechanism of oGvHD especially, a greater need to understand how the meibomian glands are affected by aHSCT and their involvement in oGvHD using suitable pre-clinical models together with sound research strategies. Collectively, knowledge about how the meibomian glands and other ocular structures are affected can inform better therapeutic strategies for treating oGvHD patients in the future.

## 9. Literature Search Strategy

We performed careful search of literature from the Medline/PubMed, Google Scholar, Scopus and Cochrane Library databases. We employed a sensitive search strategy where the key search terms were *“Graft-vs-host disease”* and *“GvHD”*. This was cross-referenced with terms such as ocular GvHD, ocular graft-vs-host disease, meibomian gland dysfunction, MGD, pre-clinical, animal models, MHC, MiHAs, chemotherapy, radiotherapy, cornea damage, eyelids damage, lacrimal gland damage, immunological response, and inflammation. Citations and full-text papers were exported to Endnote X6 citation manager. Relevant papers focused on ocular involvement in GvHD and published in English with clearly defined study methods were included. Since there are lack of pre-clinical models of oGvHD, we planned to include papers that were published in the last 15 years. Other papers cited in this manuscript were reviewed based on a general understanding of clinical GvHD and oGvHD pathology and therapeutic modalities.

## Figures and Tables

**Table 1:** Summary of pre-clinical animal models of ocular graft-vs-host disease and their key ocular findings.

Study	Purpose	Model Description	Pre-Conditioning Regimen	Main Ocular Findings				Therapy/OutCome
				Cornea	Conjunctiva	Lacrimal Gland	Meibomian Gland	
[22]	To develop a novel topical antifibrotic treatment against ocular chronic GVHD using vitamin A-coupled liposomes containing short interfering RNA against heat shock protein 47 (VA-lip HSP47) eye drops.	Minor histocompatibility-antigen mismatched mouse model Donor: B10.D2 (H-2^d^) recipient: BALB/c (H-2^d^)	Total body irradiation	No information	No information	Infiltration of HSP47^+^ fibroblasts Increased fibrosis Increased collagen deposition	No information	Instillation of VA-lip HSP47 agent reduced fibrosis, collagen deposition and restored normal levels of tear production
[18]	To investigate the role of ocular surface glycocalyx and mucins in graft versus host disease (GVHD)-associated dry eye and ameliorative effect of topical rebamipide, a mucin secretagogue, on GVHD-associated dry eye	Major histocompatibility class I mismatch Donor: C57BL/6 Ly 5.2+ Recipients: B6D2F1 (F1)	Total body irradiation	Presence of dry eye phenotype with keratopathy indicated by significant punctate and plaque corneal staining Reduced glycocalyx density and thickness Reduced Muc 1 and M4 and increased Muc 16	Decrease in palpebral conjunctiva goblet cells	Reduced tear film volume with concomitant reduction in Muc5ac	No information	Instillation of 2% rebamipide in balance salt solution vehicle twice daily (left eye only) attenuated reduction in tear production and corneal damage
[45]	To evaluate the efficacy of entospletinib (ENTO), tyrosine kinase SYK inhibitor on the clinical (clinical and skin) aspects of GVHD	Donor: C57BL/6 (H2b) Recipient: BALB/c (H2kd) Clarification on matching status need	Total body irradiation	No specific information	Chemosis, redness	No specific information	Eyelid edema and blepharitis but no direct mention of the meibomian glands	ENTO administration resulted in profound improvements in clinical eye as well as other systemic GvHD scores.
[21]	To develop a novel clinical scoring criterion for identifying degrees of ocular pathology at both the ocular surface and adnexa in oGvHD	MHC-matched, minor transplantation antigen–mismatched allogeneic model of matched unrelated donor Donors: B6 mice (H-2^b^, Thy1.1)//(eGFP) B6 transgenic (H2^b^) Recipients: C3H.SW (H2^b^)	Total body irradiation	Corneal ulceration, epithelial haze, corneo-limbal eGFP^+^ immune cell infiltrates	No information	No specific information	Eye lid oedema and closure with eGFP^+^ immune cell infiltrates but no specific mention of the meibomian gland	No information
[42]	To identify the kinetics and origin of ocular infiltrating T cells in a preclinical model of graft-versus-host disease (GVHD) that induces eye tissue damage.	Major histocompatibility complex-matched, minor histocompatibility-mismatched hematopoietic stem cell transplant mouse model. Donor: C56BL/6 mice (H2^b^, Ly9.1^−^) Recipient: C3H.SW, H2^b^, Ly9.1^+^	Total body irradiation	Epitheliopathy with increased staining 3–4wks post-transplant Infiltration of CD4+ and CD8+ T cells, macrophages, monocytes and neutrophils Upregulation of IFNy, TNFα and IL-6 genes	Reduced goblet cells density Apparent atrophy of fornix	Infiltration of T cells and macrophages	No information	No information
[20]	To establishes a model of GVHD with cornea and limbus involvement	Major/sex mismatch and Donor: Male C57BL/6 (H2b) Recipient: Female BALB/c (H2k)	Total body irradiation	Atrophic epitheliopathy with vacuolization Stromal oedema, neovascularization, and inflammatory/lymphocytic infiltrates Limbus show epithelial satellitosis	Focal epithelial loss, lymphocytic exocytosis, necrosis Mononuclear cells and microvesicular infiltrates	Apparent ductal fibrosis Ductal/Interlobu-lar infiltration with eosinophils	Crusted/erythe-matosus eyelids but no direct mention of the meibomian gland	No information
[19]	To describe lacrimal gland involvement in graft versus-host disease	Major histocompatibility class I mismatch Donors: C57BL/6 Ly 5.2+ (Allogeneic), B6D2F1 (F1) (Syngeneic) Recipients: B6D2F1 (F1)	Total body irradiation	No information	No information	Periductal inflammation fibrosis, apoptosis, accumulation of ductal debris and stasis of ducts Infiltration of of CD3+, CD8+ and CD4+ positive T cells Overall higher disease scores in the allogeneic group	Lid inflammation but no direct mention of the meibomian gland involvement	No information

## Abbreviations

aHSTC	Allogeneic hematopoietic stem cell transplantation
CD	Cluster of Differentiation
GvHD	Graft-vs-host disease
HLA	Human leukocyte antigen
MHC	Major histocompatibility complex
MiHA	Minor histocompatibility antigen
MDA	Malondialdehyde
MGD	Meibomian glad dysfunction
oGvHD	Ocular graft-vs-host-disease
